# The complete mitochondrial genome of the firefly *Curtos fulvocapitalis* (Coleoptera: Lampyridae)

**DOI:** 10.1080/23802359.2021.1958080

**Published:** 2021-12-13

**Authors:** Wei Li, Quan Liu, Xinhua Fu

**Affiliations:** a Institute of Artificial Intelligence, Jianghan University, Wuhan, China; b Hubei Insect Resources Utilization and Sustainable Pest Management Key Laboratory, College of Plant Science and Technology, Huazhong Agricultural University, Wuhan, China; c Firefly Conservation Research Centre, Wuhan, China

**Keywords:** *Curtos fulvocapitalis*, mitochondrial genome, phylogenetic relationship, evolutionary relationship

## Abstract

We report the complete mitochondrial genome of the firefly *Curtos fulvocapitalis* Jeng et Yang 1998. The circular genome is 16,398 bp and has a base composition of A (42.21%), C (11.22%), G (7.73%), and T (38.85%). Our sequence is similar to those of other metazoans, which contain 13 protein-coding genes. All 13 protein-coding genes were initiated by the ATN (ATT, ATA, and ATG) codon. Nine protein-coding genes stopped with TAA or TAG codons, and the other four genes had an incomplete termination codon, a single T. We sequenced the mitochondrial genome of fireflies to analyze phylogenetic relationships and determine the evolution of their flashing signals.

The genus Curtos of Lampyridae includes 19 species in southeastern Asia (Fu et al. 2012). Only *C. costipennis* (Gorham), *C. mundulus* (Olivier), *C. sauteri* (Olivier), and *C. sp* were found in mainland China before 2014 (Fu 2014). In addition, we found that two mitochondrial genomes of this genus have been reported: *C. costipennis* (Zhang and Fu 2019) and *C. bilineatus* (Chen et al. 2019). In this paper, we focus on the MtDNA of *C. fulvocapitalis* and attempt to analyze the phylogenetic relationship and evolutionary relationship of Curtos fireflies.

Specimens used in this study were collected from Taxia Village, Zhangzhou City, Fujian Province (117°3′E, 24°36′N) and were stored at the Natural History Museum, Huazhong Agricultural University, Wuhan City, Hubei Province (http://www.hzau.edu.cn/, Xinhua Fu, fireflyfxh@mail.hzau.edu.cn) under voucher number CF2017062201.

Whole-body genomic DNA was extracted from each individual using the Aidlab Genomic DNA Extraction Kit (Aidlab Co., Beijing, China) following the manufacturer’s protocols.

Primers were designed according to the mitochondrial genomic sequences of closely related species, and long PCR (LA-PCR) amplification was performed using LA Taq polymerase. The PCR conditions were as follows: initial denaturation at 98 °C for 2 min, then 40 cycles of denaturation at 98 °C for 10 s, annealing at 50 °C for 15 s, and extension at 68 °C for 1 min/kb, followed by a final extension at 68 °C for 10 min.

After quality-checking the obtained fragments, the complete mitochondrial genome sequence was assembled manually using DNAstar v7.1 software (Burland 2000). The MtDNA was annotated roughly following the procedure described previously by Zou et al. and Zhang et al. (Peng et al. 2017; Zhang et al. 2018).

The precise boundaries of rrnL and rrnS were determined via a comparison with homologs. PhyloSuite (Zhang et al. 2018), an in-house GUI-based software, was used to parse and extract the information from genomes that were manually annotated in Word documents and to create GenBank submission files and organizational tables for mitogenomes. Genomic statistics of the two diplectanids, as well as all available monogeneans, were also extracted by PhyloSuite.

Twenty mitogenomic sequences (downloaded from GenBank) were used in the phylogenetic analysis, which was performed using the MrBayes method with PhyloSuite programs (Figure 1).

**Figure 1.**
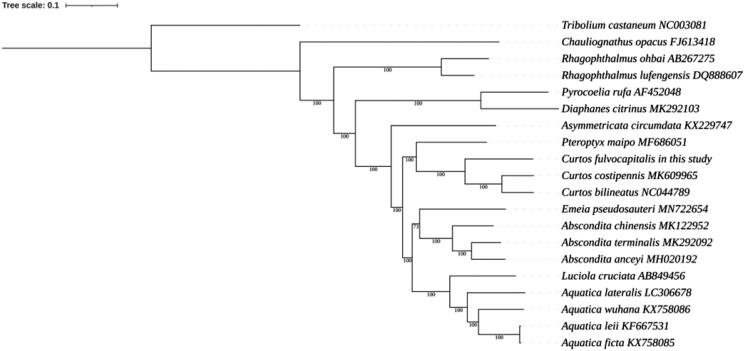
Molecular phylogenetic analysis of the complete mitochondrial genome by the MrBayes method.

The complete mitochondrial genome sequence of *C. fulvocapitalis* (GenBank: MW582616) is 16,398 bp and has a base composition of A (42.21%), C (11.22%), G (7.73%), and T (38.85%). Our sequence is similar to those of other metazoans, which contain 13 protein-coding genes, 22 tRNA genes, 2 rRNA genes, and a noncoding AT-rich region. All 13 PCGs started with ATN (ATT, ATA, and ATG) codons. Among those genes, six PCGs (COII, COIII, ND4, ND4L, ATP6, and CTYB) started with ATG, two PCGs (ND3 and ND6) started with ATT, and five PCGs (COI, ND5, ND1, ND2, and ATP8) started with ATA. In addition, an incomplete stop codon, namely, a single T codon, was found in four PCGSs (COII, COIII, ND5, ND4). In the case of the nine other PCGSs, TAAs (ND2, ND6, ATP8, ATP6, ND4L, COI) or TAGs (CTYB, ND1, ND3) were used.

The phylogenetic relationship analysis (Figure 1) shows that *C. fulvocapitalis* is most closely related to *C. bilineatus* and *C. costipennis*, which belong to the genus Curtos.

Muraji et al performed a phylogenetic analysis of the partial COI and 28S sequences of *C. costipennis* and *C. okinawanus* distributed on the Ryukyu Islands of Japan (Muraji et al. 2012). However, this analysis did not completely reflect the evolutionary relationships of Curtos fireflies. To examine the evolutionary relationships of Curtos fireflies, (i) we should obtain more samples of Curtos fireflies from mainland China, Taiwan Province and Japan, and (ii) we should study the evolutionary relationship based on the complete mitochondrial genome of these fireflies. In this paper, the complete mitochondrial genome sequence of *C. fulvocapitalis* provides important insight into the evolution of Curtos fireflies in eastern Asia.

## Data Availability

The data that support the findings of this study are openly available at the US National Center for Biotechnology Information (NCBI database) at https://www.ncbi.nlm.nih.gov/. The GenBank accession No. (reference number) is MW582616. The corresponding author’s departments are Huazhong Agricultural University College of Plant Science and Technology and Firefly Conservation Research Center. The ranking of departments is that the first department is Huazhong Agricultural University College of Plant Science and Technology, and the second department is the Firefly Conservation Research Center.
